# 1,3-Dipolar Cycloaddition in the Preparation of New Fused Heterocyclic Compounds via Thermal Initiation

**DOI:** 10.3390/molecules21020187

**Published:** 2016-02-04

**Authors:** Martin Porubský, Lukáš Tenora, Milan Potáček

**Affiliations:** Department of Chemistry, Masaryk University, Kotlářská 2, Brno 611-37, Czech Republic; 380165@mail.muni.cz (M.P.); 175258@mail.muni.cz (L.T.)

**Keywords:** 1,3-dipolar cycloaddition, fused heterocycles, thermal initiation, 3-amino-benzo[*b*]furan-2-carbaldehyde

## Abstract

This paper describes the synthesis of precursors with a benzo[*b*]furan skeleton for the intramolecular 1,3-dipolar cycloaddition of azomethine ylides prepared from *N*-substituted 3-allyl-aminobenzo[*b*]furan-2-aldehydes and secondary amines derived from α-amino acid esters. Reactions were initiated by heating. The products consisted of four fused rings with three stereogenic centers. Their structure and stereochemistry were determined by NMR spectra and X-ray measurements.

## 1. Introduction

**Scheme 1:**
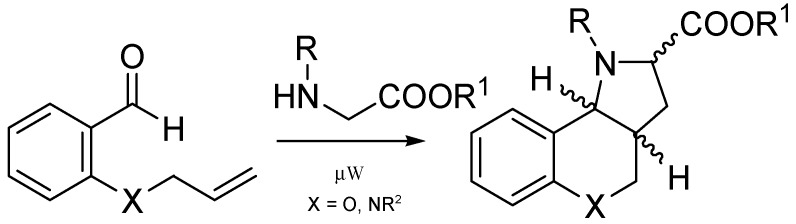
Microwave-initiated 1,3-dipolar cycloadditions leading to hexahydrochromeno[4,3-*b*] pyrroles and hexahydropyrrolo[3,2-*c*]quinolines.

The 1,3-dipolar cycloaddition reaction (1,3-DC) is a powerful tool for the synthesis of five-membered heterocyclic compounds. The reactions are characterized by high regio- and stereoselectivity. For the reaction, a dipole and a dipolarophile are required. There are several types of dipoles which can participate in cyclization reactions. In the case of a molecule containing both assemblies of atoms, one representing a dipole and the other a dipolarophile, it is possible to achieve intramolecular 1,3-DC and, in this way, the formation of a new ring [1,2,3]. One frequently used method for preparing azomethine ylides applies an aldehyde and an amino-substituted ester. Such a method was used in our laboratory and, under microwave irradiation, led to hexahydrochromeno[4,3-*b*]pyrroles [4,5,6] and hexahydropyrrolo[3,2-*c*]quinolines [7]. The procedure is shown in Scheme 1.

**Scheme 2:**
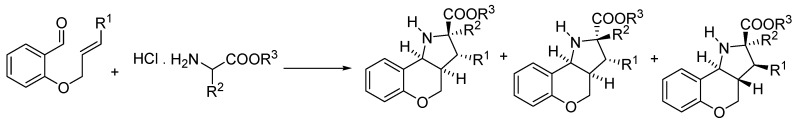
Overview of 1,3-DC products prepared in [8] and their configuration.

In a recent paper, Cossío [8] presented a large experimental study of the formation of chromeno-[4,3-*b*]pyrrolidines, which also included a computational assessment of origin of the 1,3-DC stereocontrol. The authors prepared starting aldehydes by means of the *O*-alkylation of salicylaldehyde with cinamyl bromide according to a standard procedure. The partners in the 1,3-DC reactions were α-aminocarboxylic acid ester hydrochlorides (Scheme 2) and the cyclizations were carried out using both microwave irradiation (neat, 185 °C) and conventional heating in polyethylene glycol (170 °C). Three diastereomers of the expected cycloaddition products were found, as determined by GC-MS and ^1^H-NMR of the crude products. A significant part of the paper was devoted to computational analysis of the intramolecular cycloaddition.

All the fused heterocyclic compounds mentioned above demonstrated important physiological properties. The pyrrolidino[3,2-*c*]tetrahydroquinoline skeleton is found in the natural alkaloid martinelline and martinellic acid, isolated from tropical plant roots, both of which exhibit biological activity as antagonists of the bradykinine B_1_ and B_2_ receptors and antibiotic activity [9]. Similarly, the tetrahydrochromeno[4,3-*b*]pyrrolidine structure is found to be a constituent of many compounds exibiting activity as non-competitive antagonists of the muscular nicotinic acetylcholine receptor and the muscarinic acetylcholine receptor [10]. It is also present in various natural compounds such as sceletium alkaloid [11], and in synthetically prepared forms, displays activity against several bacterial pathogens [12].

Moreover, many experiments carried out on biological material have shown that the benzo[*b*]furan skeleton present in both synthesized compounds as well as products isolated from natural sources also demonstrates various biological activities. Thus, the insecticidal, fungicidal, cytotoxic and antioxidative properties of naturally occurring benzo[*b*]furan derivatives isolated from plants of the *Styracaceae* [13] and *Moraceae* [14] families have been examined. Similar activity has also been found in synthetic materials [15,16,17]. Benzo[*b*]furan systems in combination with a chromone or cumarine structure displayed also antimicrobial, antiviral, anticancer, anti-inflammatory, and antioxidant properties [18]. With that knowledge and our experience with the application of intramolecular 1,3-dipolar cycloaddition, we decided to extend our synthesis to more complex systems containing benzo[*b*]furan skeletons.

## 2. Results and Discussion

**Scheme 3:**
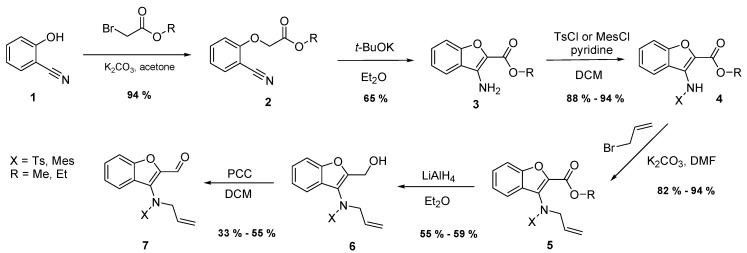
Reaction path leading to the synthesis of the key starting molecule *N*-allyl-*N*-(2-formyl-benzo[*b*]furan-3-yl)-4-methylbenzene sulfonamide (**7**) used for the generation of azomethine ylides.

Here, for the task to build new systems we had to prepare starting molecules. The necessary key starting molecule was *N*-substituted, *N*-allyl-3-aminobenzo[*b*]furan-2-carbaldehyde (**7**). The procedure applied in its preparation is depicted in Scheme 3.

The synthesis began with commercially available 2-hydroxybenzonitrile (**1**), which was used as a nucleophile for the substitution of bromine in the alkyl bromoacetate molecule and for the preparation of alkyl-(2-cyanophenoxy) acetate **2**. Optimal conditions for the formation of the carbanion from **2** and its nucleophilic attack leading to cyclic compound **3** were sought by varying the solvent, base, and temperature. The application of *t*-butoxide in diethyl ether at room temperature for 25 min was identified as the optimal procedure. In the following step, it was necessary to introduce an allyl group to the already present free amino group. However, before doing so, it was necessary to protect the amino group from diallylation. Therefore, we introduced both tosyl or mesyl groups by the application of the corresponding sulfonylchloride in an excess of pyridine in dichloromethane at room temperature overnight. The following treatment with allyl bromide in the presence of potassium carbonate in DMF at room temperature for about 20 min led from compound **4** to amine **5** via the introduction of the allyl group.

The procedure leading to key molecule **7** for 1,3-dipolar cycloadditions containing an aldehyde group included one intermediate product, **6**. Instead of the direct transformation of ester **5** to aldehyde **7**, for instance by DIBAL, which appeared to have limited success, we achieved the transformation in two steps. The first reductive step was carried out by means of the ester **5** reaction with LiAlH_4_. Our best results were characterized by yields of alcohol **6** approaching 55%. For the following oxidation step, we tested pyridinium chlorochromate in dichloromethane and found it to be successful.

**Scheme 4:**
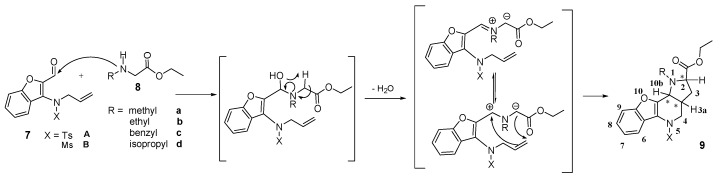
Plausible mechanism of azomethine ylide formation from compound **7** and ethyl-*N*-substituted 2-amino acetate (**8**), and its immediate intramolecular 1,3-dipolar cycloaddition to the already present double bond to form fused heterocycle **9**.

Intramolecular 1,3-dipolar cycloaddition is based upon the formation of a 1,3-dipole *in situ* in a molecule that contains a double bond in a position which is capable of interacting with the dipole [4]. In our case, we generated azomethine ylides from compound **7** by its reaction with ethyl-*N*-alkyl-2-amino acetates (**8**). The probable mechanism of the reaction is presented in Scheme 4.

In the reaction, the nucleophilic secondary amine attacks the electrophilic carbon atom of the aldehyde; after water splits off, the 1,3-dipole—azomethine ylide—is formed *in situ*. This participates in an intramolecular 1,3-DC and a new heterocyclic system is formed. During the addition, three stereogenic centres are created in the new molecule (Scheme 4) at positions *2*, *3a* and *10b*. Thus, the existence of four possible diastereoisomers should be expected. However, even when the temperature of the reaction was varied and the reaction mixtures were analyzed by LC/MS, LC/UV and NMR spectra, only one major diastereoisomer was found. 

In all cases, reactions were carried out without any solvent in a bath preheated to 130–140 ° C. Aldehyde **7** dissolved immediately after being put into the corresponding ester **8**. It was found that doubling the amount of ester **8** increased the product yield. The reaction time required for consumption of the whole amount of compound **7** ranged from 20 to 35 min depending upon the temperature and the steric requirements of the *N*-alkyl substitution. The reaction products were isolated by HPFC, with the EtOAc/PE mobile phase ratio equal to 1:3.

**Figure 1:**
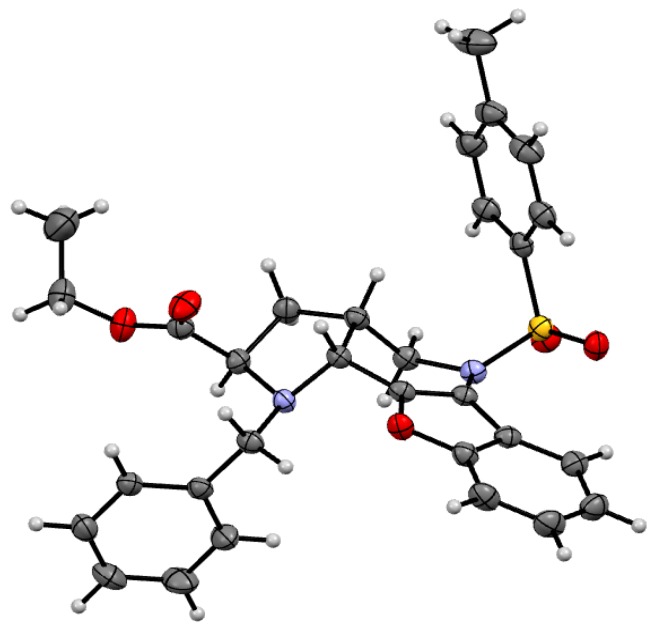
ORTEP diagram of molecule **9Ac**.

The NOESY spectrum indicated that the hydrogen atoms at *10b* and *3a* were oriented towards one side and to the side opposite to the hydrogen atom at *2*. This finding was supported by observation of the hydrogen atoms at *3* and *3*′. NOESY interactions were observed only between the hydrogen atoms at *2* and *3*′ and between those at *3*′′ and *3a*, which indicates the opposite orientation of the hydrogen atoms at the *2* and *3a* positions (Supplementary Materials). All these conclusions were confirmed by X-ray structure analysis (Figure 1).

**Figure 2:**
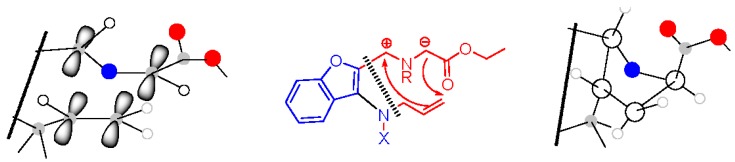
Perspective view of the ylide fragment conformation affording diastereomer **9** with the configuration (2R*, 3aR*, 10b*) and a schematic view of the product. In the scheme we neglect the plus/minus sign of the wave function; the interactions have a stabilizing effect.

Figure 2 attempts to depict, in perspective view, the conformation of the *in situ*-formed ylide entering 1,3-dipolar cycloaddition with the configuration of the products observed.

## 3. Experimental Section

### 3.1. General Information

All chemicals were used as purchased. If necessary, solvents were dried according to literature procedures [19]. DCM was distilled from anhydrous CaCl_2_, then from P_2_O_5_, and stored over MS (3 Å). Et_2_O was dried over Na and freshly distilled before use. Pyridine was distilled from KOH and stored over MS (3 Å). 2-Hydroxybenzonitrile (**1**) was purchased from Sigma Aldrich (St. Louis, MO, USA).

Most reactions were carried out under a dry argon atmosphere and all reactions were monitored by TLC (F254 silica gel, Merck, Darmstadt, Germany). Column chromatography was performed on a Horizon HPFC system (Biotage, Uppsala, Sweden) equipped with a FLASH Si 25 + M cartridge. Melting points were measured on an MPM HV2 instrument (Kleinfeld Labortechnik GmbH, Gehrden, Germany). FT-IR spectra were recorded on a Genesis ATI (Unicam, Cambridge, UK) apparatus. ^1^H- and ^13^C-NMR spectra were recorded using an Avance 300 spectrometer (Bruker, Karlsruhe, Germany) operating at frequencies of 300.13 MHz (^1^H) and 75.47 MHz (^13^C) with CDCl_3_ as the solvent. Tetramethylsilane (δ = 0.00 ppm) or CHCl_3_ (δ = 7.27 ppm) served as internal standards for ^1^H-NMR spectra, and CDCl_3_ (δ = 77.23 ppm) for ^13^C-NMR spectra. GC-MS data were obtained on a GCMS-QP2010 (Shimadzu, Kyoto, Japan) in EI mode at 30 eV. MS data were obtained on a TRIO 1000 spectrometer (Fisons Instruments, Hazlet, NJ, USA) at 30 eV in electron impact mode and by thermal desorption. High Resolution Mass Spectra (HRMS) were recorded on a 6224 Accurate-Mass TOF LC-MS (Agilent, DE, Wilmington, WI, USA) in APCI and ESI modes. X-ray diffraction data were collected on a KM-4 four-circle CCD diffractometer (Kuma, Wroclaw, Poland) and corrected for Lorentz and polarization effects. The structures were resolved by direct methods and refined by full-matrix least-squares methods using the SHELXTL program package [20]. Hydrogen atoms were placed in calculated idealized positions and refined as riding. The crystal structure was deposited at CCDC [21].

### 3.2. Chemistry

*Methyl-(2-cyanophenoxy)acetate* (**2**). Potassium carbonate (2.78 g, 20.2 mmol, 1.20 eq.) and methyl bromoacetate (3.08 g, 20.2 mmol, 1.20 eq.) were added to a solution of 2-hydroxybenzonitrile (**1**, 2.00 g, 16.8 mmol, 1.00 eq.) dissolved in dried acetone (85 mL). The reaction mixture was refluxed for 7 h. After cooling down to room temperature, the mixture was filtered and the acetone solution evaporated. The reaction afforded light yellow crystals with a yield of 94%; mp 69 °C. ^1^H-NMR: δ = 7.59 (dd, *J* = 7.7, 1.6, 1H, H1), 7.52 (td, *J* = 9.2, 7.7, 1.7, 1H, H4), 7.07 (td, *J* = 7.6, 0.7, 1H, H2), 6.86 (d, *J* = 8.5, 1H, H3), 4.78 (s, 2H, CH_2_), 3.81 (s, 3H, COOCH_3_). ^13^C-NMR: δ = 168.6 (COOCH_3_), 159.8 (C6), 134.3 and 134.5 (C2 and C3), 122.2 (C1), 116.3 (C5), 112.7 (C4), 103.0 (CN), 66.0 (CH_2_), 52.7 (COOCH_3_). MS (EI, 70 eV) Found: 191 (M calculated for: C_10_H_9_NO_3_ 191.19); *m*/*z* (%) = 191 (M^+^, 50), 162 (50), 146 (10), 132 (70), 117 (15), 102 (70), 85 (60), 83 (100), 75 (35), 45 (60).

*Methyl-3-aminobenzo[b]furan-2-carboxylate* (**3**). Compound **2** (2.62 g, 13.9 mmol, 1.00 eq.) was dissolved in dried diethyl ether and poured into a flask in which an argon atmosphere was maintained. Then, potassium *t*-butoxide (0.78 g, 6.9 mmol, 0.50 eq.) was carefully added and the mixture stirred at room temperature. After 25 min, several drops of water were added and the solvent evaporated. The remainder was mixed with water (30 mL) and the mixture extracted three times with 30 mL of diethyl ether. After drying by sodium sulfate, filtration, and the evaporation of ether, light yellow crystals were obtained with a yield of 65%; mp 94 °C. ^1^H-NMR: δ = 7.55 (d, *J* = 7.9, 1H, H4), 7.48–7.41 (m, 2H, H7 and H6), 7.19–7.30 (m, 1H, H5), 5.00 (bs, 2H, NH_2_), 3.96 (s, 3H, COOCH_3_). ^13^C-NMR: δ = 162.1 (COOCH_3_), 154.3 (C7a), 138.9 (C3), 129.1 (C4), 125.6 (C3a), 122.5 (C5), 121.8 (C2), 119.8 (C6), 112.8 (C7), 51.7 (COOCH_3_). MS (EI, 70 eV) Found: 191 (calculated for C_10_H_9_NO_3_: 191.19); *m*/*z* (%) = 191 (M^+^, 100), 159 (50), 133 (25), 103 (90), 83 (20), 77 (50), 51 (25).

*General procedure for the preparation of compounds* **4A** *and* **4B**. Either tosyl chloride (1.29 g, 6.78 mmol, 1.20 eq.) or methane sulfonylchloride (1.29 g, 11.3 mmol, 2.00 eq.) were added to a stirred solution of **3** (1.16 g, 5.65 mmol, 1.00 eq.) and pyridine (1.35 g, 16.9 mmol, 3.00 eq.) in dichloromethane (12 mL), and the mixture was stirred under an argon atmosphere at room temperature overnight. The progress of the reaction was monitored by TLC. After removal of the solvent, the crude product was dissolved in ethyl acetate (40 mL) and subsequently extracted by 3 × 20 mL water, 1 × 15 mL HCl, and 1 × 15 mL brine. The extract was dried by sodium sulfate, filtered, and then thickened to crystallization. It was then subsequently recrystallized either from ethyl acetate or acetone.

*Methyl-3-tosylaminobenzo[b]furan-2-carboxylate* (**4A**), colourless crystals, yield 88%, mp 116 °C. ^1^H-NMR: δ = 8.36 (bs, 1H, NH), 8.31 (d, 1H, *J* = 8.1, H4), 7.60 (d, *J* = 8.2, 2H, H_Ts_), 7.41–7.54 (m, 2H, H7 and H6), 7.37 (t, *J* = 7.4, 1H, H5), 7.17 (d, *J* = 8.2, 2H, H_Ts_), 3.86 (s, 3H, COOCH_3_), 2.35 (s, 3H, SO_2_C_6_H_4_CH_3_). ^13^C-NMR: δ = 161.0 (COOCH_3_), 154.3 (C7a), 144.5 (C3), 135.3 (C tosyl), 132.7 (C tosyl), 130.4 (C2), 129.8 (2C tosyl), 129.4 (C6), 127.6 (2C tosyl), 124.4 (C4), 124.3 (C5), 122.4 (C3a), 112.5 (C7), 52.5 (COOCH_3_), 21.7 (NHSO_2_C_6_H_4_CH_3_). MS (EI, 70 eV) Found: 345 (calculated for C_17_H_15_NO_5_S: 345.37); *m*/*z* (%) = 345 (M^+^, 50), 222 (10), 190 (45), 162 (40), 134 (55), 119 (20), 103 (20), 91 (100), 76 (30), 65 (45).

*Methyl-3-mesylaminobenzo[b]furan-2-carboxylate* (**4B**) ochre crystals, yield 94%, mp 173 °C. ^1^H-NMR: δ = 8.24 (bs, 1H, NH), 8.19 (d, *J* = 8.2, 1H, H4), 7.53 (d, *J* = 3.6, 2H, H7 and H6), 7.34–7.41 (m, 1H, H5), 4.03 (s, 3H, COOCH_3_), 3.09 (s, 3H, SO_2_CH_3_). ^13^C-NMR: δ = 161.5 (COOCH_3_), 154.5 (C7a), 132.2 (C3), 129.6 (C6), 124.4 (C4), 124.1 (C5), 122.5 (C2), 122.0 (C3a), 112.6 (C7), 52.8 (COOCH_3_), 40.3 (NHSO_2_CH_3_). MS (EI, 70 eV) found: 269 (calculated for C_11_H_11_NO_5_S: 269.27); *m*/*z* (%) = 269 (M^+^, 70), 237 (15), 190 (85), 162 (50), 148 (15), 134 (100), 119 (30), 103 (50), 91 (35), 76 (70), 59 (20), 50 (25).

*General procedure for the preparation of compounds* **5A** *and* **5B**. A solution of compound **4A** or **4B** (3.24 mmol, 1.00 eq.) in DMF (10 mL) was mixed with anhydrous potassium carbonate (672 mg, 4.86 mmol, 1.50 eq.). While the mixture was stirred, allyl bromide (590 mg, 4.86 mmol, 1.50 eq.) was added dropwise. The stirring continued for 20 h (for compound **5A**) and 6 h (for **5B**). The progress of the reaction was monitored by TLC (EtOAc/PE = 1:2). The process was finished by removing the solvent and the remainder was mixed with water (20 mL); the final product was extracted by dichloromethane and the extract washed with brine (15 mL). After drying by Na_2_SO_4_, filtration, and removal of the solvent by distillation, the product was crystallized from methanol.

*Methyl-3-(N-allyl-N-tosylamino)benzo[b]furan-2-carboxylate* (**5A**) white crystals, yield 94%, mp 86 °C. ^1^H-NMR: δ = 7.64 (d, *J* = 8.2, 2H, H_Ts_), 7.53 (d, 1H, *J* = 8.3, H4), 7.43–7.50 (m, 2H, H7 and H6), 7.27–7.36 (m, 1H, H5), 7.27 (d, *J* = 8.2, 2H, H_Ts_), 5.75–5.92 (m, 1H, CH_2_CH=CH_2_), 5.05 (dd, *J* = 17.1, 1.2, 1H, CH_2_CH=CH_2_), 4.99 (dd, *J* = 10.1, 0.9, 1H, CH_2_CH=CH_2_), 4.10–4.56 (bs, 2H, CH_2_CH=CH_2_), 3.67 (s, 3H, COOCH_3_), 2.43 (s, 3H, SO_2_C_6_H_4_CH_3_). ^13^C-NMR: δ = 158.8 (COOCH_3_), 154.0 (C7a), 144.0 (C3), 141.3 (C tosyl), 136.7 (C tosyl), 133.3 (CH_2_CH=CH_2_), 129.8 (2C tosyl), 128.7 (C6), 128.0 (2C tosyl), 127.7 (C2), 127.4 (C3a), 124.6 (C4), 122.6 (C5), 119.5 (CH_2_CH=CH_2_), 112.7 (C7), 54.3 (CH_2_CH=CH_2_), 52.5 (COOCH_3_), 21.9 (SO_2_C_6_H_4_CH_3_). MS (EI, 70 eV) Found: 230 [M − Ts] (calculated for C_20_H_19_NO_5_S: 385.434); *m*/*z* (%) = 230 (100), 198 (85), 170 (45), 115 (25), 91 (45), 65 (20), 45 (30).

*Methyl-3-(N-allyl-N-mesylamino)benzo[b]furan-2-carboxylate* (**5B**) orange crystals, yield 82%, mp 91 °C. ^1^H-NMR: δ = 7.80 (d, *J* = 7.8, 1H, H4), 7.44–7.56 (m, 2H, H7 and H6), 7.33–7.40 (m, 1H, H5), 5.72–5.90 (m, 1H, CH_2_CH=CH_2_), 5.11 (dd, *J* = 17.1, 1.3, 1H, CH_2_CH=CH_2_), 5.02 (dd, *J* = 10.1, 1.2, 1H, CH_2_CH=CH_2_), 4.40 (bs, 2H, CH_2_CH=CH_2_), 4.02 (s, 3H, COOCH_3_), 3.06 (s, 3H, SO_2_CH_3_). ^13^C-NMR: δ = 159.5 (COOCH_3_), 154.0 (C7a), 140.4 (C3), 133.2 (CH_2_CH=CH_2_), 129.1 (C6), 128.9 (C2), 127.7 (C3a), 124.9 (C4), 122.6 (C5), 119.7 (CH_2_CH=CH_2_), 112.7 (C7), 54.1 (CH_2_CH=CH_2_), 52.9 (COOCH_3_), 40.1 (SO_2_CH_3_). MS (EI 70 eV) Found: 230 [M − Ms] (calculated for C_14_H_15_NO_5_S: 309.338); *m*/*z* (%) = 230 (80), 198 (100), 170 (70), 156 (15), 115 (30), 102 (15), 83 (70), 59 (15), 45 (45).

*Ester group reduction—transformation of compound* **5** *to compound* **6**. A solution of compound **5A** or **5B** (1.27 mmol, 1.00 eq.) in dry diethyl ether (13 mL) was slowly added to an intensively stirred suspension of LiAlH_4_ (58 mg, 1.5 mmol, 1.2 eq.) in dry diethyl ether (2 mL) cooled down to 0 °C under an argon atmosphere. After addition, the temperature was raised to room temperature and the reaction mixture was kept at this temperature for a further 30 min. Then, the reaction was stopped by the addition of diethyl ether containing several drops of water. Subsequently, the reaction mixture was extracted by diethyl ether (3 × 10 mL). The collected extracts were dried by Na_2_SO_4_ and filtered, and the solvent was further evaporated. The liquid product was purified by column chromatography (EtOAc/PE = 1:3).

*N-(2-Hydroxymethylbenzo[b]furan-3-yl)-N-allyl-4-methylbenzenesulfonamide* (**6A**) light yellow oil, yield 59%. ^1^H-NMR: δ = 7.54 (d, *J* = 8.2, 2H, H_Ts_), 7.42 (d, *J* = 8.3, 1H, H4), 7.12–7.24 (m, 3H, H6 and 2H_Ts_), 6.87–6.98 (m, 1H, H5), 6.41 (d, *J* = 7.8, 1H, H7), 5.72–5.91 (m, 1H, CH_2_CH=CH_2_), 5.03 (dd, *J* = 16.9, 1.2, 1H in CH_2_CH=CH_2_), 5.05 (dd, *J* = 1.0, *J* = 10.1, 1H in CH_2_CH=CH_2_), 4.19–4.88 (bs, 4H, 2H in CH_2_CH=CH_2_ and 2H in CH_2_OH), 2.41 (s, 3H, SO_2_C_6_H_4_CH_3_). ^13^C-NMR: δ = 157.1 (C7a), 153.7 (C2), 144.4 (C3), 135.9 (C tosyl), 132.6 (CH_2_CH=CH_2_), 130.0 (2C tosyl), 127.8 (2C tosyl), 125.1 (C6), 124.2 (C tosyl), 122.9 (C5), 119.9 (C4), 119.1 (CH_2_CH=CH_2_), 117.4 (C3a), 112.5 (C7), 55.3 (CH_2_OH), 52.9 (CH_2_CH=CH_2_), 21.8 (SO_2_C_6_H_4_CH_3_). HRMS (ESI) (*m*/*z*): [M + H]^+^ calculated for C_19_H_20_NO_4_S^+^: 358.1108. Found: 385.1112.

*N-(2-Hydroxymethylbenzo[b]furan-3-yl)-N-allyl-methanesulfonamide* (**6B**) light yellow oil, yield 55%. ^1^H-NMR: δ = 7.54 (d, *J* = 7.9, 1H, H4), 7.44–7.49 (m, 1H, H7), 7.33–7.41 (m, 1H, H5), 7.31 (dd, *J* = 1.2, 7.4, 1H, H6), 5.78–5.96 (m, 1H, CH_2_CH=CH_2_), 5.06–5.18 (m, 2H, CH_2_CH=CH_2_), 4.70 (s, 2H, CH_2_OH), 4.37 (bs, 2H, CH_2_CH=CH_2_), 2.99 (s, 3H, NHSO_2_CH_3_). ^13^C-NMR: δ = 157.3 (C7a), 154.0 (C2), 132.6 (CH_2_CH=CH_2_), 125.7 (C6), 124.5 (C3), 123.9 (C5), 120.3 (C4), 118.6 (CH_2_CH=CH_2_), 116.9 (C3a), 113.1 (C7), 55.3 (CH_2_OH), 53.2 (CH_2_CH=CH_2_), 39.5 (NHSO_2_CH_3_). HRMS (ESI) (*m*/*z*): [M + H]^+^ calculated for C_13_H_16_NO_4_S^+^: 282.0795. Found: 282.0795.

*Procedure for the preparation of compound* **7**. Pyridinium chlorochromate (400 mg, 1.86 mmol, 2.50 eq.) was added to a solution of alcohol **6** (0.74 mmol, 1.00 eq.) in dry dichloromethane (5 mL). The resulting black suspension was stirred under argon at room temperature for 26 h. Subsequently, diethyl ether (15 mL) was added and the suspension stirred again for 10 min. Then, the upper layer was decanted and filtered through a short column (5 cm) of Florisil. The obtained light yellow solution was concentrated to crystallization and the product recrystallized from ethanol.

*N-Allyl-N-(2-formylbenzo[b]furan-3-yl)-4-toluenesulfonamide* (**7A**) yellow crystals, yield 55%, mp 107 °C. ^1^H-NMR: δ = 9.72 (s, 1H, CH=O), 7.58 (d, *J* = 8.2, 2H, H_Ts_), 7.56 (d, *J* = 7.6, 1H, H4), 7.43–7.51 (m, 1H, H6), 7.20–7.29 (m, 2H, H_Ts_), 7.18 (t, *J* = 7.5, 1H, H5), 7.08 (d, *J* = 7.8, 1H, H7), 5.73–5.89 (m, 1H, CH_2_CH=CH_2_), 5.06 (dd, *J* = 9.4, 0.9, 1H in CH_2_CH=CH_2_), 5.04 (dd, *J* = 17.0, 0.9, 1H in CH_2_CH=CH_2_), 4.35 (bs, 2H, CH_2_CH=CH_2_), 2.43 (s, 3H, SO_2_C_6_H_4_CH_3_). ^13^C-NMR: 179.0 (CH=O), 161.1 (C7a), 155.0 (C3), 149.4 (C-tosyl), 144.8 (C2), 135.2 (C-tosyl), 132.0 (CH_2_CH=CH_2_), 130.1 (2C tosyl), 129.6 (C6), 127.9 (2C tosyl), 124.7 (C3a), 124.8 (C5), 122.0 (C4), 120.6 (CH_2_CH=CH_2_), 113.5 (C7), 53.8 (CH_2_CH=CH_2_), 21.8 (SO_2_C_6_H_4_CH_3_). MS (EI 70 eV): Found: 200 [M − Ts] (calculated for C_19_H_17_NO_4_S: 355.408); *m*/*z* (%) = 200 (100), 172 (60), 158 (30), 144 (25), 115 (20), 103 (15), 91 (95), 77 (15), 65 (50).

*N-Allyl-N-(2-formylbenzo[b]furan-3-yl)-methanesulfonamide* (**7B**) yellow oil, yield 33%. ^1^H-NMR: δ = 10.02 (s, 1H, CH=O), 7.80 (d, *J* = 8.0, 1H, H4), 7.52–7.62 (m, 2H, H7 and H6), 7.37–7.44 (m, 1H, H5), 5.72–5.89 (m, 1H, CH_2_CH=CH_2_), 5.11 (dd, *J* = 17.1, 1.2, 1H, CH_2_CH=CH_2_), 5.08 (dd, *J* = 10.1, 0.9, 1H, CH_2_CH=CH_2_), 4.43 (d, *J* = 0.7, 2H, CH_2_CH=CH_2_), 3.02 (s, 3H, NSO_2_CH_3_). ^13^C-NMR: 180.5 (CH=O), 155.0 (C7a), 148.1 (C3), 133.7 (C2), 132.3 (CH_2_CH=CH_2_), 130.0 (C6), 125.1 (C5), 122.4 (C4), 120.4 (CH_2_CH=CH_2_), 118.1 (C3a), 113.4 (C7), 53.6 (CH_2_CH=CH_2_), 39.4 (NSO_2_CH_3_). HRMS (APCI): *m*/*z* [M + H]^+^ calculated for C_13_H_13_NO_4_S^+^: 280.0638. Found: 280.0638.

*General procedure for the formation of azomethine ylide and its following intramolecular 1,3-dipolar cycloaddition—preparation of compound* **9**. Compound **7** (0.14 mmol, 1.00 eq.) and a two-fold excess of secondary amine **8** (0.28 mmol, 2.00 eq.) were mixed in a test tube filled with an argon atmosphere and stopped with a balloon. Then, the tube was immersed in a preheated oil bath (130–140 °C) and stirred for about 30 min until the mixture became darker in color. After cooling down, the mixture was separated by HPFC (AcOEt/PE = 1:3). The products were crystallized from methanol.


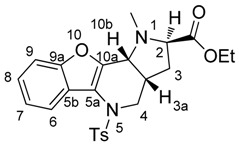


*(2R,3aR,10bR) and (2S,3aS,10bS)-Ethyl-1-methyl-5-tosyl-2,3,3a,4,5,10b-hexahydro-1H-benzo[b] furo[3,2-b]pyrrolo[2,3-d]pyridine-2-carboxylate* (**9Aa**). Yellow crystals, yield 80%, mp 64 °C. ^1^H-NMR: δ = 8.12–8.14 (m, 1H, H6), 7.49 (d, *J* = 8.3, 2H, H_Ts_), 7.44 (dd, *J* = 1.7, 7.1, 1H, H9), 7.28–7.35 (m, 2H, H8 and H7), 7.19 (d, *J* = 8.0, 2H, H_Ts_), 4.10–4.18 (m, 2H, COOCH_2_CH_3_), 4.07 (dd, *J* = 14.1, 4.8, 1H, H4), 3.73 (d, *J* = 6.7, 1H, H10b), 3.64 (dd, *J* = 2.1, 8.6, 1H, H2), 3.17 (dd, *J* = 14.0, 12.0, 1H, H4), 2.63 (s, 3H, NCH_3_), 2.39 (s, 3H, SO_2_C_6_H_4_CH_3_), 2.03–2.09 (m, 1H, H3), 1.95–2.02 (m, 1H, H3a), 1.62–1.68 (m, 1H, H3), 1.25 (t, *J* = 7.1, 3H, COOCH_2_CH_3_). ^13^C-NMR: δ = 173.3 (COOCH_2_CH_3_), 153.9 (C9a), 148.1 (C10a), 144.1 (C5a), 135.3 (C-SO_2_C_6_H_4_CH_3_), 129.8 (2CH SO_2_C_6_H_4_CH_3_), 127.4 (2CH-SO_2_C_6_H_4_CH_3_), 124.9 (C8), 123.2 (C SO_2_C_6_H_4_CH_3_), 123.1 (C6), 122.8 (C7), 119.1 (C5b), 111.3 (C9), 65.0 (C2), 60.3 (COOCH_2_CH_3_), 56.0 (C10b), 51.0 (C4), 36.4 (NCH_3_), 33.5 (C3a), 31.7 (C3), 21.6 (SO_2_C_6_H_4_CH_3_), 14.3 (COOCH_2_CH_3_). MS (EI, 70 eV) found 454 (calculated for C_24_H_26_N_2_O_5_S: 454.54); *m*/*z* (%) = 454 (M^+^, <5), 381 (65), 299 (20), 225 (100), 211 (15), 196 (10), 184 (10), 170 (30), 155 (10), 91 (55), 65 (20), 57 (45). HRMS (ESI) *m*/*z* [M + H]^+^ calculated for C_24_H_27_N_2_O_5_S^+^: 455.1635). Found: 455.1636.


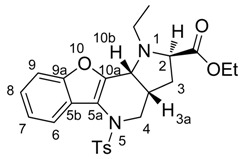


*(2R,3aR,10bR) and (2S,3aS,10bS)-Ethyl-1-ethyl-5-tosyl-2,3,3a,4,5,10b-hexahydro-1H-benzo[b] furo[3,2-b]pyrrolo[2,3-d]pyridine-2-carboxylate* (**9Ab**). Light yellow crystals, yield 94%, mp 105 °C. ^1^H-NMR: δ = 8.13–8.16 (m, 1H, H6), 7.48 (d, *J* = 8.3, 2H, H_Ts_), 7.44 (dd, *J* = 1.7, 7.1, 1H, H9), 7.28–7.34 (m, 2H, H8 and H7), 7.19 (d, *J* = 8.1, 2H, H_Ts_), 4.10–4.17 (m, 2H, COOCH_2_CH_3_), 4.05 (dd, *J* = 14.1, 4.7, 1H, H4), 3.86 (d, *J* = 6.7, 1H, H10b), 3.81 (dd, *J* = 1.9, 8.7, 1H, H2), 3.30–3.38 (m, 1H, NCH_2_CH_3_), 3.21 (dd, *J* = 14.0, 11.8, 1H, H4), 2.64–2.72 (m, 1H, NCH_2_CH_3_), 2.38 (s, 3H, SO_2_C_6_H_4_CH_3_), 2.03–2.08 (m, 1H, H3), 1.92–2.00 (m, 1H, H3a), 1.61–1.67 (m, 1H, H3), 1.25 (t, *J* = 7.1, 3H, COOCH_2_CH_3_), 1.04 (t, *J* = 7.3, 3H, NCH_2_CH_3_). ^13^C-NMR: δ = 173.6 (COOCH_2_CH_3_), 153.9 (C9a), 148.2 (C10a), 144.1 (C5a), 135.4 (C in SO_2_C_6_H_4_CH_3_), 129.8 (2CH in SO_2_C_6_H_4_CH_3_), 127.4 (2CH in SO_2_C_6_H_4_CH_3_), 124.8 (C8), 123.2 (C in SO_2_C_6_H_4_CH_3_), 123.1 (C6), 122.8 (C7), 119.1 (C5b), 112.3 (C9), 60.7 (C2), 60.2 (COOCH_2_CH_3_), 55.4 (C4), 51.0 (NCH_2_CH_3_), 43.3 (C10b), 32.9 (C3a), 31.5 (C3), 21.6 (SO_2_C_6_H_4_CH_3_), 14.3 (COOCH_2_CH_3_), 13.6 (NCH_2_CH_3_). MS (EI, 70 eV) Found: 395 [M − COOEt] (calculated for C_25_H_28_N_2_O_5_S: 468.57); *m/z* (%) = 395 (80), 313 (20), 239 (100), 211 (25), 184 (15), 170 (20), 91 (40), 71 (25), 56 (30). HRMS (ESI) *m/z* [M + H]^+^ calculated for C_25_H_29_N_2_O_5_S^+^: 469.1792). Found: 469.1791.


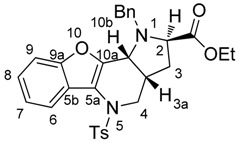


*(2R,3aR,10bR) and (2S,3aS,10bS*)-*Ethyl-1-benzyl-5-tosyl-2,3,3a,4,5,10b-hexahydro-1H-benzo[b] furo[3,2-b]pyrrolo[2,3-d]pyridine-2-carboxylate* (**9Ac**). Yellow crystals, yield 96%, mp 130 °C. ^1^H-NMR: δ = 8.17–8.21 (m, 1H, H6), 7.50 (d, *J* = 8.3, 2H, H_Ts_), 7.37–7.42 (m, 1H, H9), 7.30–7.33 (m, 2H, H8 and H7), 7.20 (d, *J* = 8.6, 2H, H_Ts_), 7.15–7.18 (m, 5H, CH_2_C_6_H_5_), 4.58 (d, *J* = 13.7, 1H, H4), 4.06–4.14 (m, 3H, 2H in COOCH_2_CH_3_ and 1H in CH_2_C_6_H_5_), 4.05 (d, *J* = 3.6, 1H, H10b), 3.48–3.51 (m, 1H, H2), 3.82 (d, *J* = 13.7, 1H, H4), 3.30 (dd, *J* = 14.0, 11.6, 1H, CH_2_C_6_H_5_), 2.40 (s, 3H, SO_2_C_6_H_4_CH_3_), 1.98–2.03 (m, 1H, H3), 1.92–1.97 (m, 1H, H3a), 1.60–1.67 (m, 1H, H3), 1.20 (t, *J* = 7.1, 3H, COOCH_2_CH_3_). ^13^C-NMR: δ = 173.8 (COOCH_2_CH_3_), 153.9 (C9a), 148.0 (C10a), 144.1 (C5a), 138.8 (C in CH_2_C_6_H_5_), 135.3 (C in SO_2_C_6_H_4_CH_3_), 129.8 (2CH in SO_2_C_6_H_4_CH_3_), 128.4 (2CH in CH_2_C_6_H_5_), 128.1 (2CH in CH_2_C_6_H_5_), 127.4 (2CH in SO_2_C_6_H_4_CH_3_), 127.0 (CH in CH_2_C_6_H_5_), 123.1 (C7), 124.9 (C8), 123.1 (C in SO_2_C_6_H_4_CH_3_), 122.9 (C6), 119.2 (C5b), 111.3 (C9), 60.5 (C2), 60.2 (COOCH_2_CH_3_), 55.1 (C10b), 52.8 (CH_2_C_6_H_5_), 51.0 (C3), 32.9 (C3a), 31.6 (C4), 21.6 (SO_2_C_6_H_4_CH_3_), 14.3 (COOCH_2_CH_3_). HRMS (APCI) *m/z* [M + H]^+^ calculated for C_30_H_31_N_2_O_5_S^+^: 531.1948). Found: 531.1947.


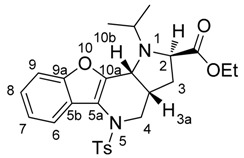


*(2R,3aR,10bR) and (2S,3aS,10bS)-Ethyl-1-isopropyl-5-tosyl-2,3,3a,4,5,10b-hexahydro-1H-benzo [b]furo[3,2-b]pyrrolo[2,3-d]pyridine-2-carboxylate* (**9Ad**). Colourless crystals, yield 44%, mp 102 °C. ^1^H-NMR: δ = 8.13–8.17 (m, 1H, H8), 7.43 (dd, *J* = 7.3, 1.4, 1H, H9), 7.27–7.34 (m, 2H, H8 and H7), 7.20 (d, *J* = 8.0, 2H, H_Ts_), 4.24 (d, *J* = 6.6, 1H, H10b), 4.05–4.13 (m, 2H, COOCH_2_CH_3_), 3.98 (dd, *J* = 14.0, 4.4, 1H, H4), 3.74 (dd, *J* = 8.5, 2.0, 1H, H2), 3.49–3.56 (m, 1H, CH(CH_3_)_2_), 3.27 (dd, *J* = 14.0, 11.1, 1H, H4), 2.39 (s, 3H, SO_2_C_6_H_4_CH_3_), 1.97–2.08 (m, 1H, H3a), 1.89–1.96 (m, 1H, H3), 1.56–1.64 (m, 1H, H3), 1.25 (t, *J* = 7.2, 3H, COOCH_2_CH_3_), 1.12 (d, *J* = 6.8, 3H, CH(CH_3_)_2_), 1.06 (d, *J* = 6.6, 3H, CH(CH_3_)_2_). ^13^C-NMR: δ = 176.6 (COOCH_2_CH_3_), 153.8 (C9a), 147.6 (C10a), 144.0 (C5a), 135.6 (C in SO_2_C_6_H_4_CH_3_), 129.8 (2CH in SO_2_C_6_H_4_CH_3_), 127.4 (2CH in SO_2_C_6_H_4_CH_3_), 124.8 (C8), 123.1 (C in SO_2_C_6_H_4_CH_3_), 123.0 (C6), 122.9 (C7), 119.3 (C5b), 111.3 (C9), 60.5 (COOCH_2_CH_3_), 58.0 (C2), 51.9 (C10b), 50.9 (C4), 47.8 (CH(CH_3_)_2_), 33.0 (C3a), 31.8 (C3), 21.6 (SO_2_C_6_H_4_CH_3_), 21.1 (CH(CH_3_)_2_), 17.8 (CH(CH_3_)_2_), 14.3 (COOCH_2_CH_3_). MS (EI 70 eV) found 482 (calculated for C_26_H_30_N_2_O_5_S: 482.59); *m/z* (%) = 482 (M^+^, <5), 409 (95), 327 (15), 253 (100), 211 (75), 184 (25), 170 (25), 155 (15), 91 (65), 84 (25), 70 (55), 65 (20), 43 (30). HRMS (ESI) *m/z* [M + H]^+^ calculated for C_26_H_31_N_2_O_5_S^+^: 483.1948). Found: 483.1948.


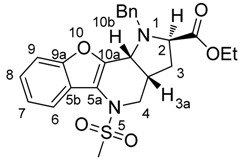


*(2R,3aR,10bR) and (2S,3aS,10bS)*-*Ethyl-1-benzyl-5-mesyl-2,3,3a,4,5,10b-hexahydro-1H-benzo[b] furo[3,2-b]pyrrolo[2,3-d]pyridine-2-carboxylate* (**9Bc**). Light yellow crystals, yield 29%, mp 106 °C. ^1^H-NMR: δ = 8.02 (d, *J* = 7.5, 1H, H6), 7.41 (d, *J* = 7.8, 1H, H9), 7.17–7.33 (m, 7H, 5H in C_6_H_5,_ H8 and H7), 4.63 (d, *J* = 13.7, 1H, CH_2_C_6_H_5_), 4.58 (d, *J* = 6.8, 1H, H10b), 4.11–4.21 (m, 2H, COOCH_2_CH_3_), 4.07 (dd, *J* = 13.6, 4.6, 1H, H4), 3.96 (d, *J* = 13.7, 1H, CH_2_C_6_H_5_), 3.61 (dd, *J* = 8.9, 1H, H2), 3.41 (dd, *J* = 13.4, 11.2, 1H, H4), 2.98 (s, 3H, NSO_2_CH_3_), 2.79–2.88 (m, 1H, H3a), 2.21–2.29 (m, 1H, H3), 1.84–1.92 (m, 1H, H3), 1.27 (t, *J* = 7.1, 3H, COOCH_2_CH_3_). ^13^C-NMR: δ = 173.7 (COOCH_2_CH_3_), 154.0 (C9a), 147.0 (C10a), 145.1 (C5a), 138.7 (C C_6_H_5_), 128.5 (2CH C_6_H_5_), 128.2 (2CH C_6_H_5_), 127.1 (CH C_6_H_5_), 125.0 (C8), 123.2 (C7), 122.2 (C6), 119.1 (C5b), 111.5 (C9), 60.8 (C2), 60.4 (COCH_2_CH_3_), 55.4 (C10b), 53.0 (CH_2_C_6_H_5_), 50.8 (C4), 39.3 (SO_2_CH_3_), 34.9 (C3a), 31.7 (C3), 14.3 (COOCH_2_CH_3_). MS (EI 70 eV) Found 381[M *−* COOEt] (calculated for C_24_H_26_N_2_O_5_S: 454.539); *m/z* (%) = 381 (40), 301 (15), 211 (20), 91 (100). HRMS (ESI) *m/z* [M + H]^+^ calculated for C_24_H_27_N_2_O_5_S^+^: 455.1641). Found: 455.1642.

## 4. Conclusions

We have prepared two *N*-substituted 3-allylaminobenzo[*b*]furan-2-aldehydes with *N*-tosyl and *N*-mesyl groups. These compounds served as precursors for the formation of azomethine ylides by means of their reaction with four types of secondary amines derived from ethyl—α-amino acid esters. Methyl, ethyl, benzyl and *iso*propyl groups were used as *N*-substituents. The ylides participated in subsequent 1,3-dipolar cycloadditions. The azomethine ylides were generated by heating a neat mixture of components to 130–140 °C. Then, they reacted intramolecularly with the already present double bond of the build in the allyl group. We found that reaction time and temperature have an influence upon the yield of cyclization rather than the size of the group bound at the amino group of the α-amino acid ester. The tosyl-protected compounds **7A** afforded products in a good yield.

Dipolar cycloaddition led to systems with four fused rings, and, during the reaction, three stereogenic centers were formed. On the basis of NOESY and 2D COSY experiments, we found compounds with (*S,S,S*) or (*R,R,R*) configurations at all centers, which was confirmed by X-ray structure analysis. These experiments proved the theory that azomethine ylide participates in cycloadditions in a more stable conformation and, therefore, that only the corresponding conformer is then found in the products.

## Supplementary Materials

The following are available online at http://www.mdpi.com/1420-3049/21/2/187/s1. Table S1: Chemical shifts of hydrogen atoms at stereogenic centres in [ppm], Table S2. Reaction conditions and product yields in 1,3-DC, Figure S1: NOESY spectrum of compound **9Ad**. ^1^H-NMR and APT spectra of final compounds **9Aa**–**d** and **9Bc** in CDCl_3_.

## References

[1] Huisgen R., Padwa A. (1984). 1,3-Dipolar Cycloaddition Chemistry.

[2] Padwa A. (1976). Intramolecular 1,3-Dipolar Cycloaddition Reactions. Angew. Chem. Int. Ed..

[3] Harwood L., Vickers R.J., Padwa A., Pearson W.H. (2003). Synthetic Applications of 1,3-Dipolar Cycloaddition Chemistry Towards Heterocycles and Natural Products.

[4] Pospíšil J., Potáček M. (2004). Microwave-assisted solvent-free synthesis of hexahydro-chromeno[4,3-*b*]pyrroles. Eur. J. Org. Chem..

[5] Pospíšil J., Potáček M. (2007). Microwave-assisted Solvent-free Intramolecular 1,3-Dipolar Cycloaddition Reactions Leading to Hexahydrochromeno[4,3-*b*] pyrroles: Scope and Limitations. Tetrahedron.

[6] Potáček M., Pospíšil J. (2004). A Solvent-free Method for Substituted Imidazolidin-4-ones Synthesis. Heterocycles.

[7] Neuschl M., Bogdal D., Potáček M. (2007). Microwave-Assisted Synthesis of Substituted Hexahydropyrrolo[3,2-*c*]quinolones. Molecules.

[8] Costa P.R.R., Sansano J.M., Cossío U., Barcellos J.C.F., Dias A.G., Nájera C., Arrieta A., Cózar A., Cossío F.P. (2015). Synthesis of Chromen[4,3-*b*]pyrrolidines by Intramolecular 1,3-Dipolar Cycloaddition of Azomethine Ylides: An Experimental and Computational Assessment of the Origin of Sterecontrol. Eur. J. Chem..

[9] Nyerges M., Fejes I., Toke L. (2000). An intermolecular 1,3-dipolar cycloaddition approach to the tricyclic core of martinelline and martinellic acid. Tetrahedron Lett..

[10] Rosini A., Budriesi R., Bixel M.G., Bolognesi M.L., Chiarini A., Hucho F., Krogsgaard-Larsen P., Mellor A., Minarini I.R., Tumiatti V. (1999). Design, synthesis, and biological evaluation of symmetrically and unsymmetrically substituted methoctramine-related polyamines as muscular nicotinic receptor noncompetitive antagonists. J. Med. Chem..

[11] Confalone P.N., Huie E.M. (1984). The Stabilized Iminium Ylide-Olefin [3+2]Cycloaddition Reaction. Total Synthesis of Sceletium Alkaloid A_4_. J. Am. Chem. Soc..

[12] Arumugam N., Raghunathan R., Almansour A.I., Karama U. (2012). An efficient synthesis of highly functionalized novel chromeno[4,3-*b*]pyrroles and indolizino[6,7-*b*]indoles as potent antimicrobial and antioxidant agents. J. Med. Chem. Lett..

[13] Choi D.H., Hwang J.W., Lee H.S., Yang D.M., Jun J.G. (2008). Highly effective total synthesis of benzofuran natural product egonol. Bull. Korean Chem. Soc..

[14] Naik R., Harmalkar D.S., Xu X., Jang K., Lee K. (2015). Bioactive benzofuran derivatives: Moracins A–Z in medicinal chemistry. Eur. J. Med. Chem..

[15] Basawaraj R., Goled S.N., Khandre O. (2011). Synthesis and biological activities of pyrazolino-benzofuro[3,2-*d*]pyrimidines. Indian J. Heterocycl. Chem..

[16] Krawiecka M., Kuran B., Kossakowski J., Cieslak M., Kazmierczak-Baranska J., Krolewska K., Nawrot B. (2015). Synthesis and Cytotoxic Properties of Halogen and Aryl-/Heteroarylpiperazinyl Derivatives of Benzofurans. Anti-cancer Agents Med. Chem..

[17] Santana L., Teijera M., Uriarte E., Teran C., Linares B., Villar R., Laguna R., Cano E. (1999). AM1 theoretical study, synthesis and biological evaluation of some benzofuran analogues of anti-inflammatory arylalkanoic acids. Eur. J. Pharm. Sci..

[18] Zwergel C., Valente S., Salvato A., Xu Z., Talhi O., Mai A., Silva A., Altucci L., Kirsch G. (2013). Novel benzofuran-chromone and -coumarine derivatives: Synthesis and biological activity in K562 human leukemia cells. Med. Chem. Commun..

[19] Armarego W.L.F., Perin D.D. (2000). Purification of Laboratory Chemicals.

[20] Sheldrick G.M. (1997). SHELXTL, Version 5.10.

[B21-molecules-21-00187] 21.CCDC 1437267 contains the supplementary crystallographic data on compound **9Ac** for this paper. Data can be obtained free of charge via http://www.ccdc.cam.ac.uk/conts/retrieving.html (or from the CCDC, 12 Union Road, Cambridge CB2 1EZ, UK; Fax: +44 1223 336033; E-mail: deposit@ccdc.cam.ac.uk).

