# Supplementary material for: 1,3-Dipolar Cycloaddition in the Preparation of New Fused Heterocyclic Compounds via Thermal Initiation
Source: Molecules. 2016 Feb 4;21(2):187. doi: 10.3390/molecules21020187 (PMC6273826; doi:10.3390/molecules21020187)
Supplement: Supplementary file 1 [file molecules-21-00187-s001.pdf]

Supplementary Materials: 1,3-Dipolar Cycloaddition in the Preparation of New Fused Heterocyclic Compounds via Thermal Initiation

Martin Porubský, Lukáš Tenora and Milan Potáček

Table S1. Chemical shifts of hydrogen atoms at stereogenic centres in δ [ppm].

Compound	H10b	H3a	H2
**9Aa**	3.73	1.65	3.64
**9Ab**	3.86	1.96	3.81
**9Ac**	4.05	1.95	3.49
**9Ad**	4.24	2.03	3.74
**9Ba**	3.73	1.99	3.64
**9Bc**	4.58	2.84	3.61

Table S2. Reaction conditions and product yields in 1,3-DC.

Compound	R	X	T [°C]	Reaction Time [min]	Yield [%]
**9Aa**	Me	Ts	140	30	80
**9Ab**	Et	Ts	130	20	34
**9Ac**	Bn	Ts	140	35	57
**9Ad**	*i*-Pr	Ts	130	20	44
**9Bc**	Bn	Ms	130	25	29

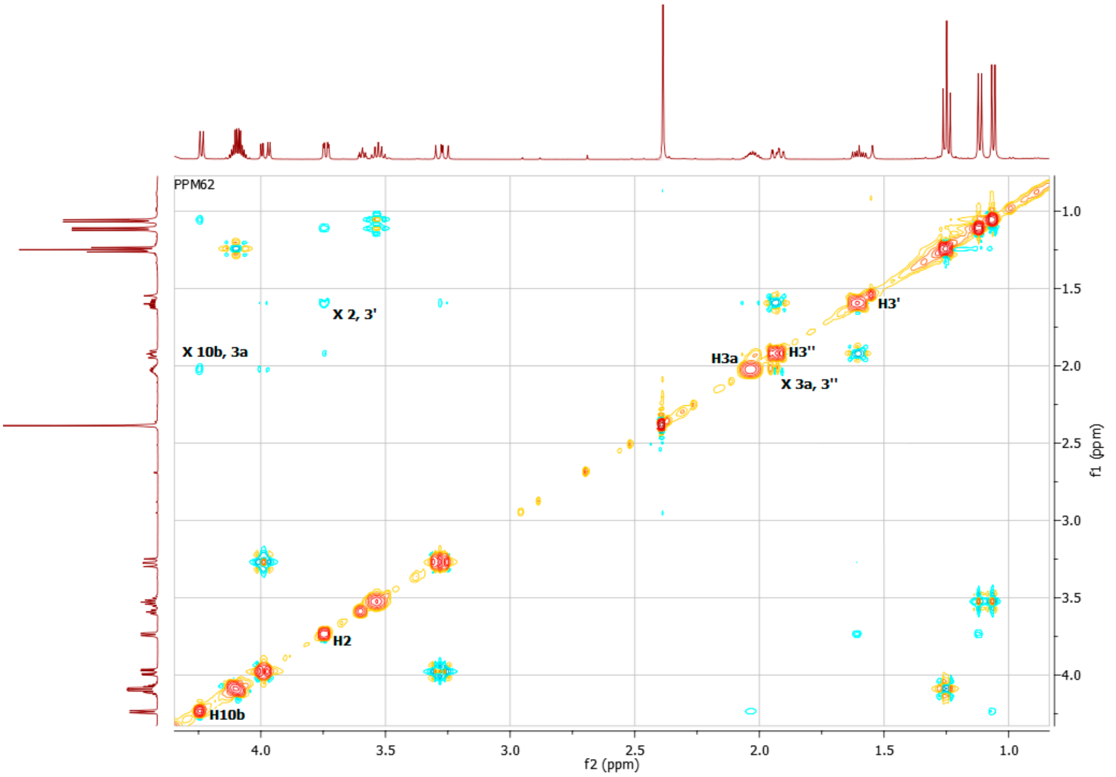

Figure S1. NOESY spectrum of compound **9Ad** (aliphatic fragment of the spectrum) picking up interactions between hydrogen atoms H10b, H3a and H2 at stereogenic centres.

^{1}H-NMR and APT Spectra of Final Compounds 9Aa–d and 9Bc in $CDCl_3$

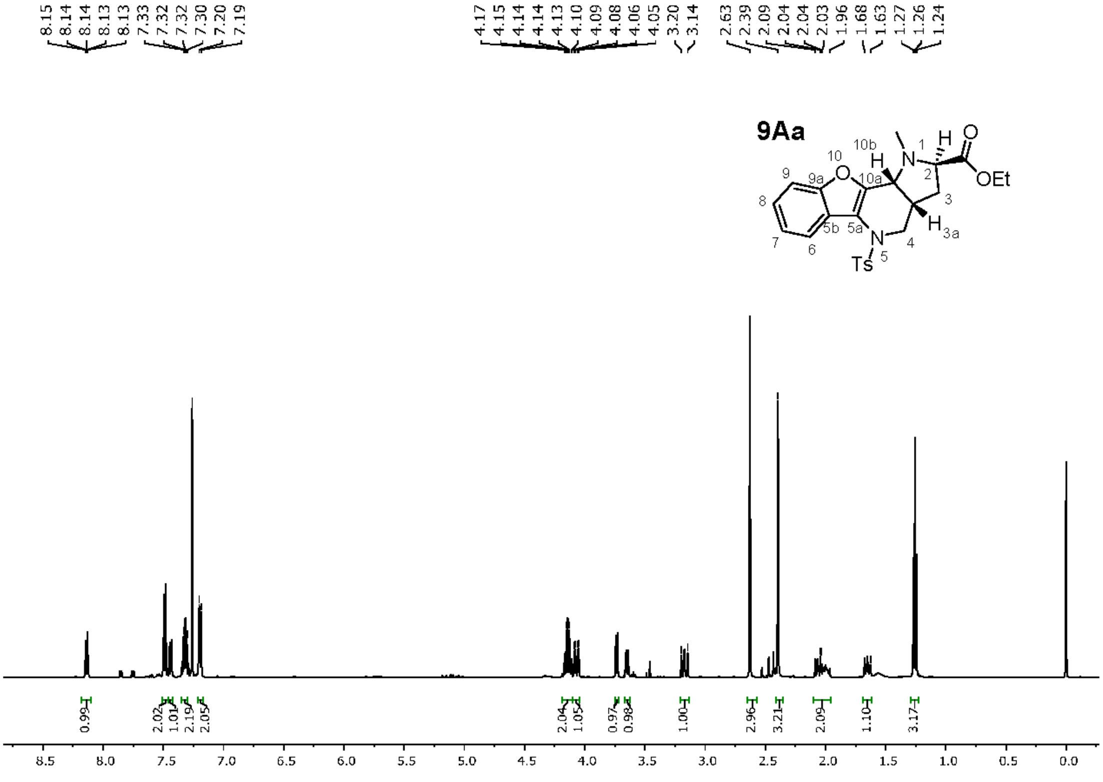

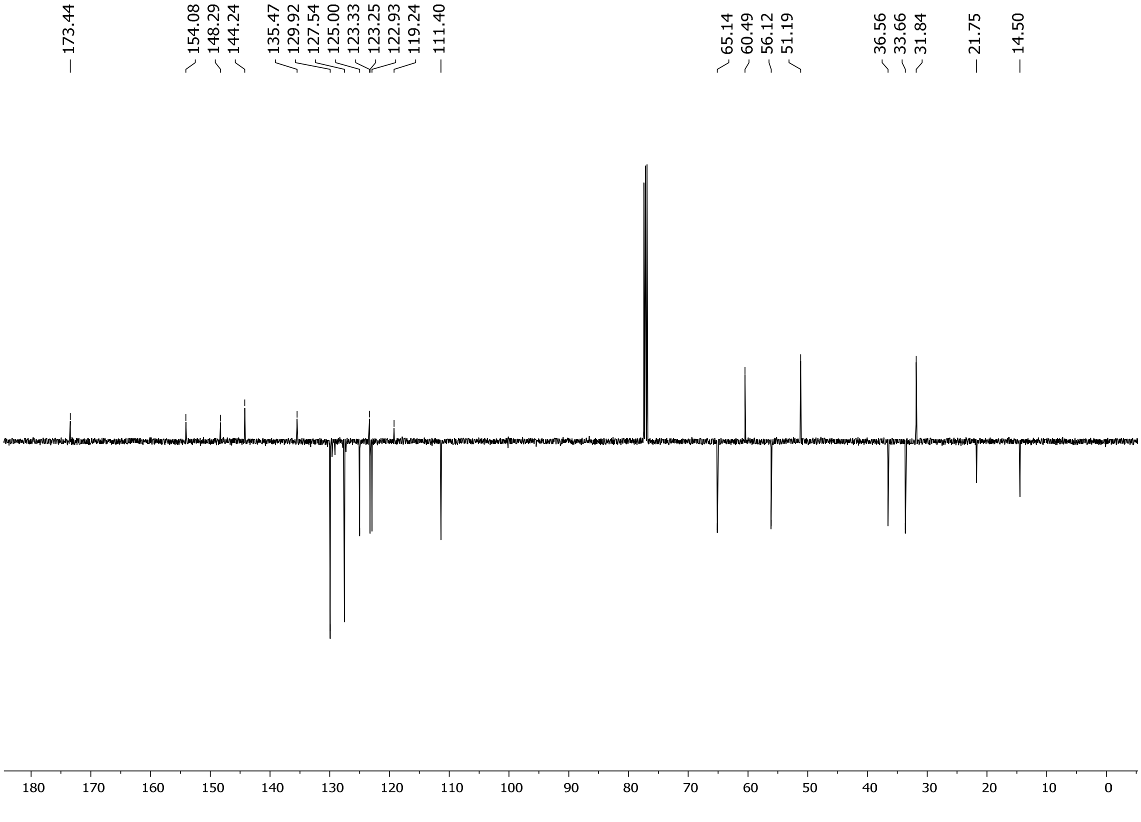

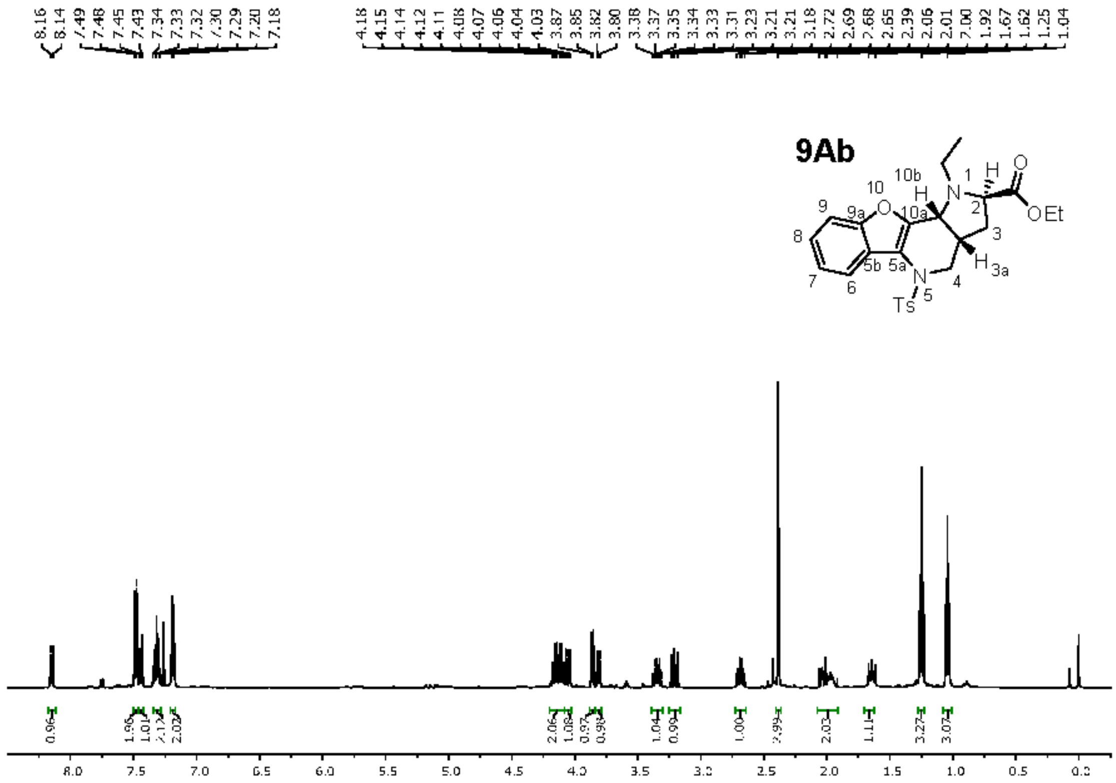
8.16 8.14 7.49 7.48 7.45 7.43 7.34 7.33 7.32 7.30 7.29 7.20 7.18
4.18 4.15 4.14 4.12 4.11 4.08 4.07 4.06 4.04 4.03 3.87 3.85 3.82 3.80 3.38 3.37 3.35 3.34 3.33 3.31 3.23 3.21 3.21 3.18 2.72 2.69 2.68 2.65 2.39 2.06 2.01 2.00 1.92 1.67 1.62 1.25 1.04
9Ab
8.0 7.5 7.0 6.5 6.0 5.5 5.0 4.5 4.0 3.5 3.0 2.5 2.0 1.5 1.0 0.5 0.0

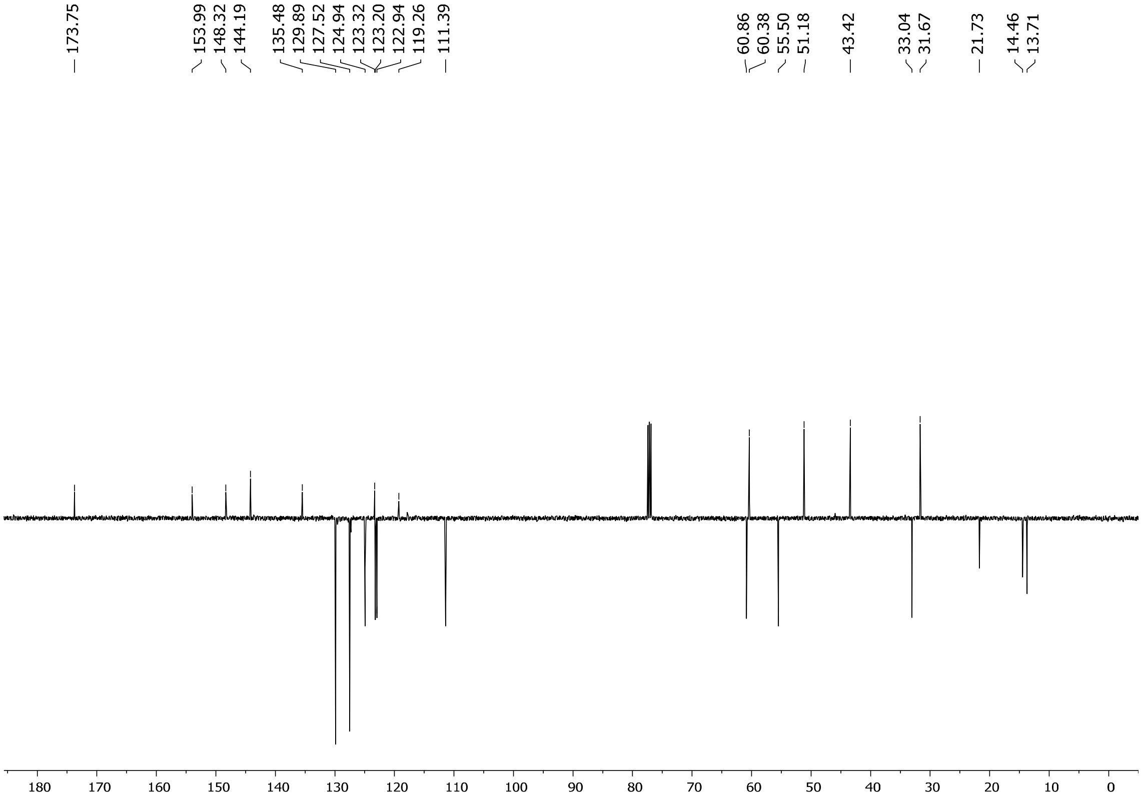
173.75
153.99 148.32 144.19
135.48 129.89 127.52 124.94 123.32 123.20 122.94 119.26 111.39
60.86 60.38 55.50 51.18
43.42
33.04 31.67
21.73
14.46 13.71
180 170 160 150 140 130 120 110 100 90 80 70 60 50 40 30 20 10 0

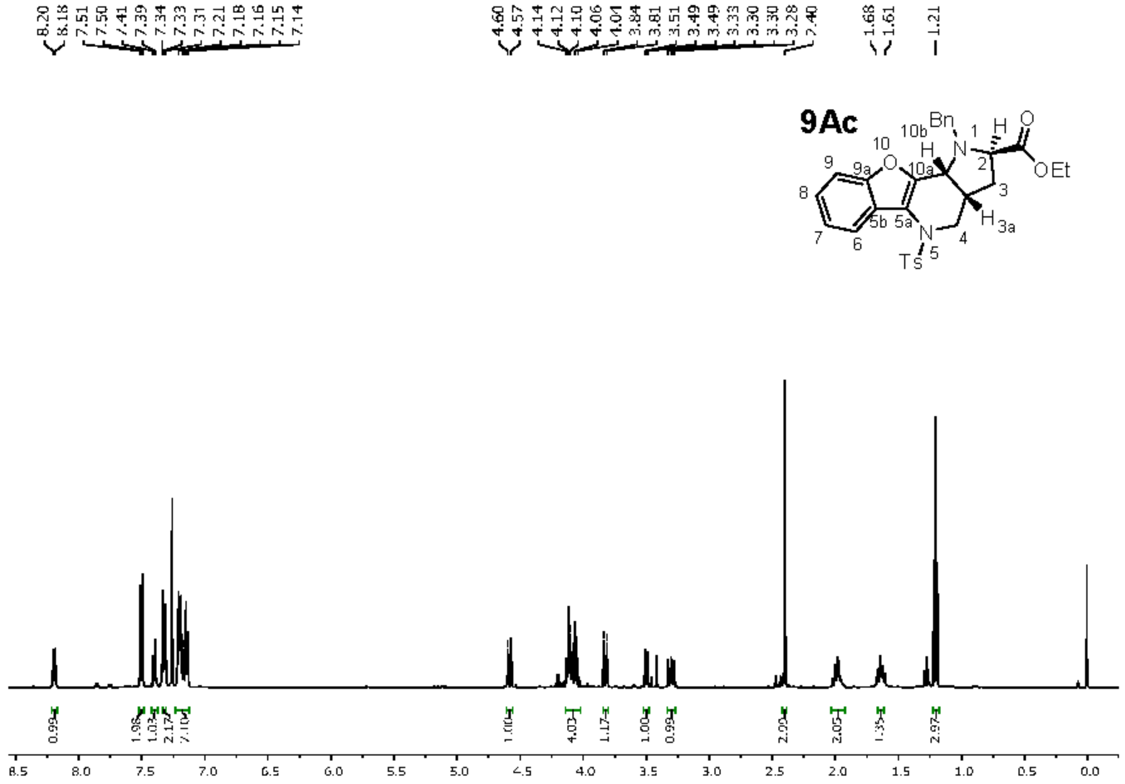
8.20 8.18 7.51 7.50 7.41 7.39 7.34 7.33 7.31 7.21 7.18 7.16 7.15 7.14
4.60 4.57 4.14 4.12 4.10 4.06 4.04 3.84 3.81 3.51 3.49 3.49 3.33 3.30 3.28 2.40
1.68 1.61 1.21
9Ac
Bn
OEt
Ts
8.5 8.0 7.5 7.0 6.5 6.0 5.5 5.0 4.5 4.0 3.5 3.0 2.5 2.0 1.5 1.0 0.5 0.0

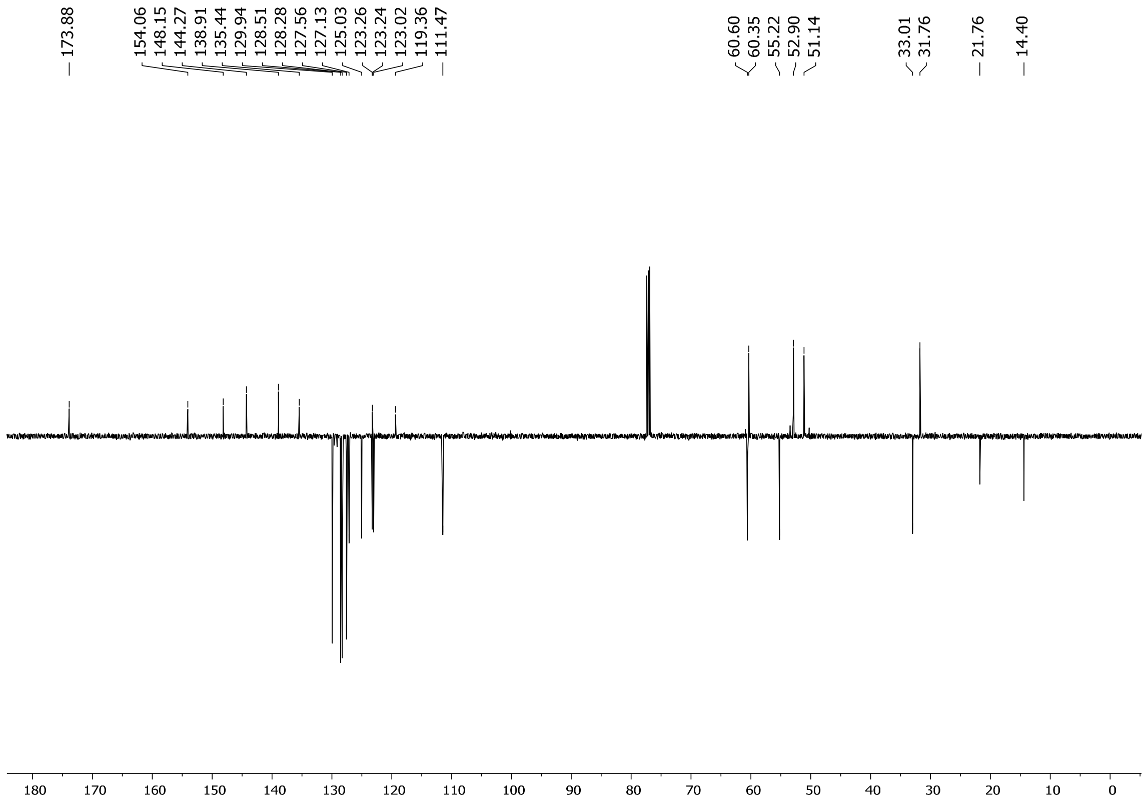
173.88
154.06 148.15 144.27 138.91 135.44 129.94 128.51 128.28 127.56 127.13 125.03 123.26 123.24 123.02 119.36 111.47
60.60 60.35 55.22 52.90 51.14
33.01 31.76
21.76
14.40
180 170 160 150 140 130 120 110 100 90 80 70 60 50 40 30 20 10 0

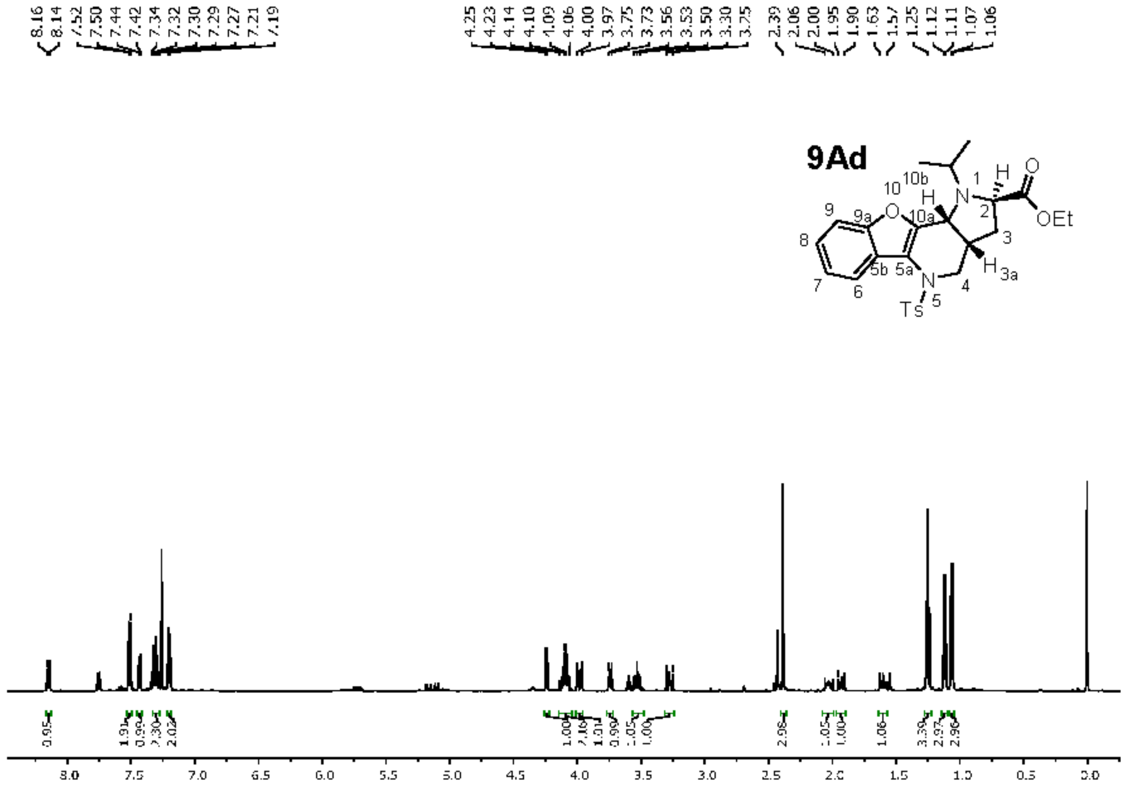
8.16
8.14
7.52
7.50
7.44
7.42
7.34
7.32
7.30
7.29
7.27
7.21
7.19
4.25
4.23
4.14
4.10
4.09
4.06
4.00
3.97
3.75
3.73
3.56
3.53
3.50
3.30
3.25
2.39
2.06
2.00
1.95
1.90
1.63
1.57
1.25
1.12
1.11
1.07
1.06
9Ad
O
OEt
H
N
Ts
0.95
1.91
0.99
2.30
2.02
1.00
1.01
0.99
1.05
1.00
2.98
1.05
1.00
1.06
3.39
2.97
2.96
8.0
7.5
7.0
6.5
6.0
5.5
5.0
4.5
4.0
3.5
3.0
2.5
2.0
1.5
1.0
0.5
0.0

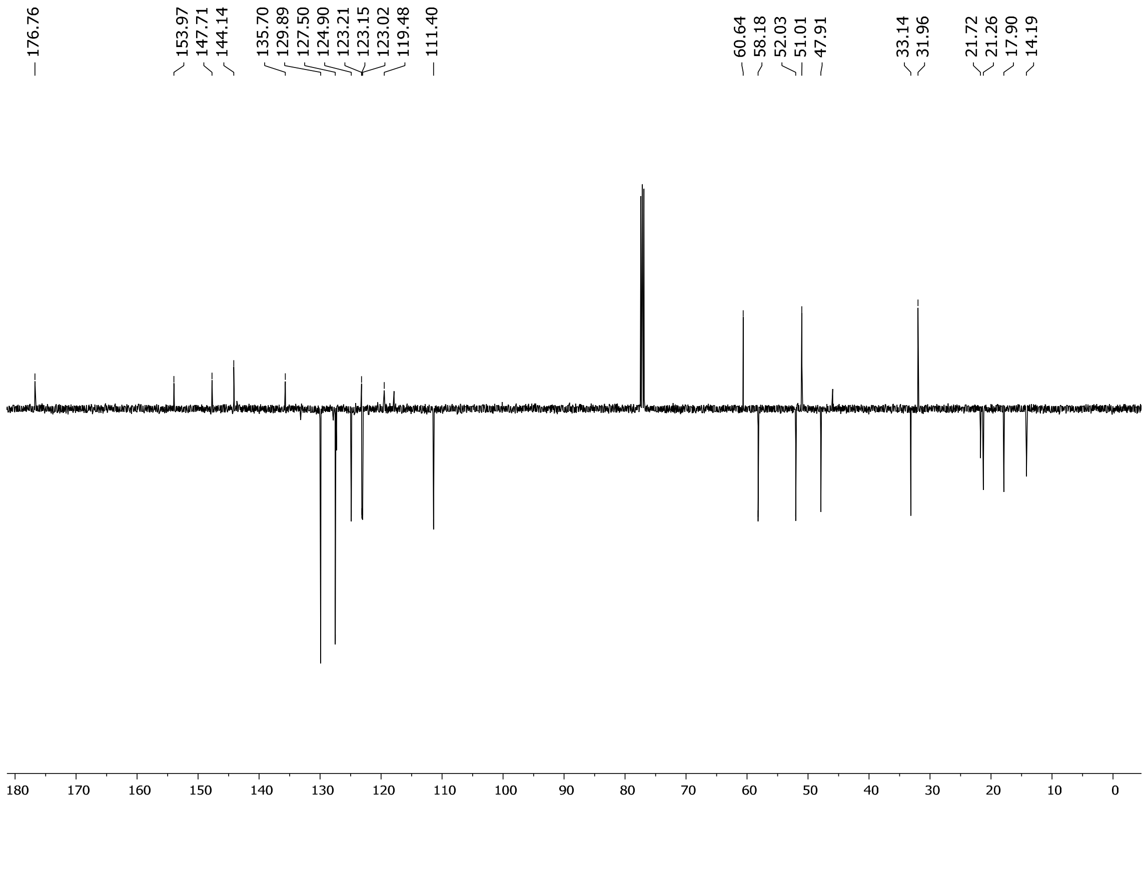
176.76
153.97
147.71
144.14
135.70
129.89
127.50
124.90
123.21
123.15
123.02
119.48
111.40
60.64
58.18
52.03
51.01
47.91
33.14
31.96
21.72
21.26
17.90
14.19
180
170
160
150
140
130
120
110
100
90
80
70
60
50
40
30
20
10
0

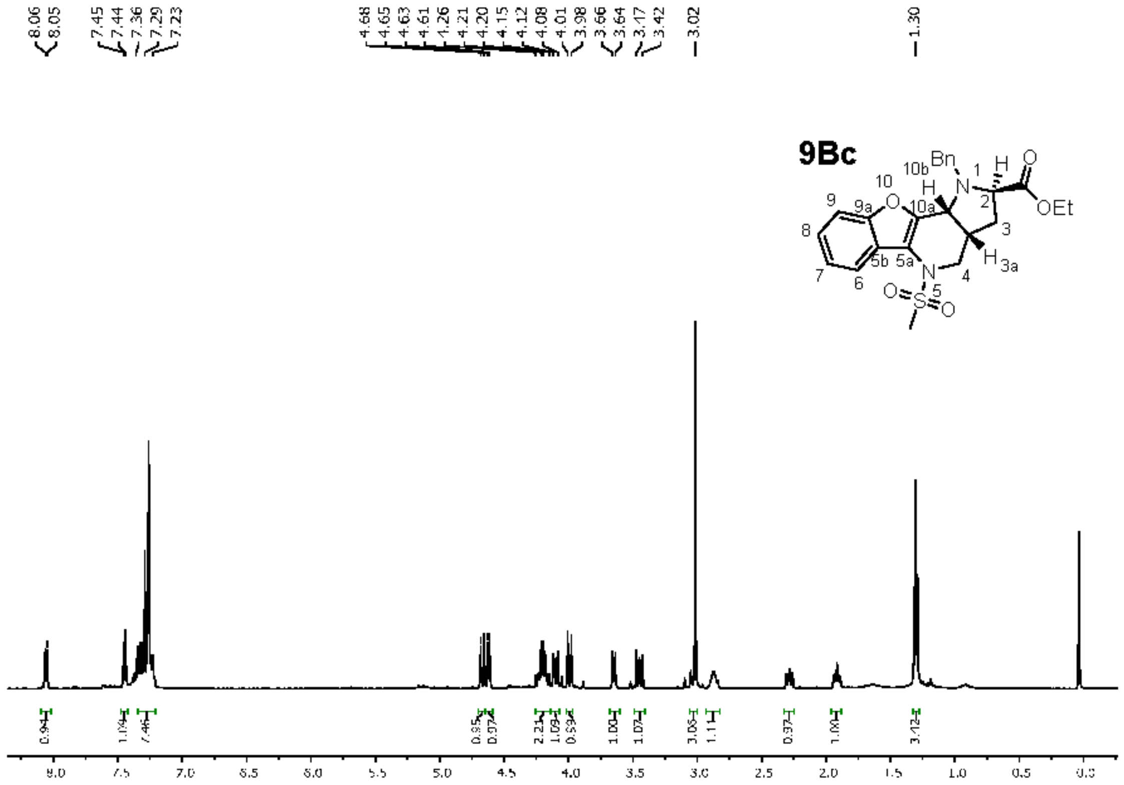
8.06
8.05
7.45
7.44
7.36
7.29
7.23
4.68
4.65
4.63
4.61
4.26
4.21
4.20
4.15
4.12
4.08
4.01
3.98
3.66
3.64
3.47
3.42
3.02
1.30
9Bc
OEt
8.0
7.5
7.0
6.5
6.0
5.5
5.0
4.5
4.0
3.5
3.0
2.5
2.0
1.5
1.0
0.5

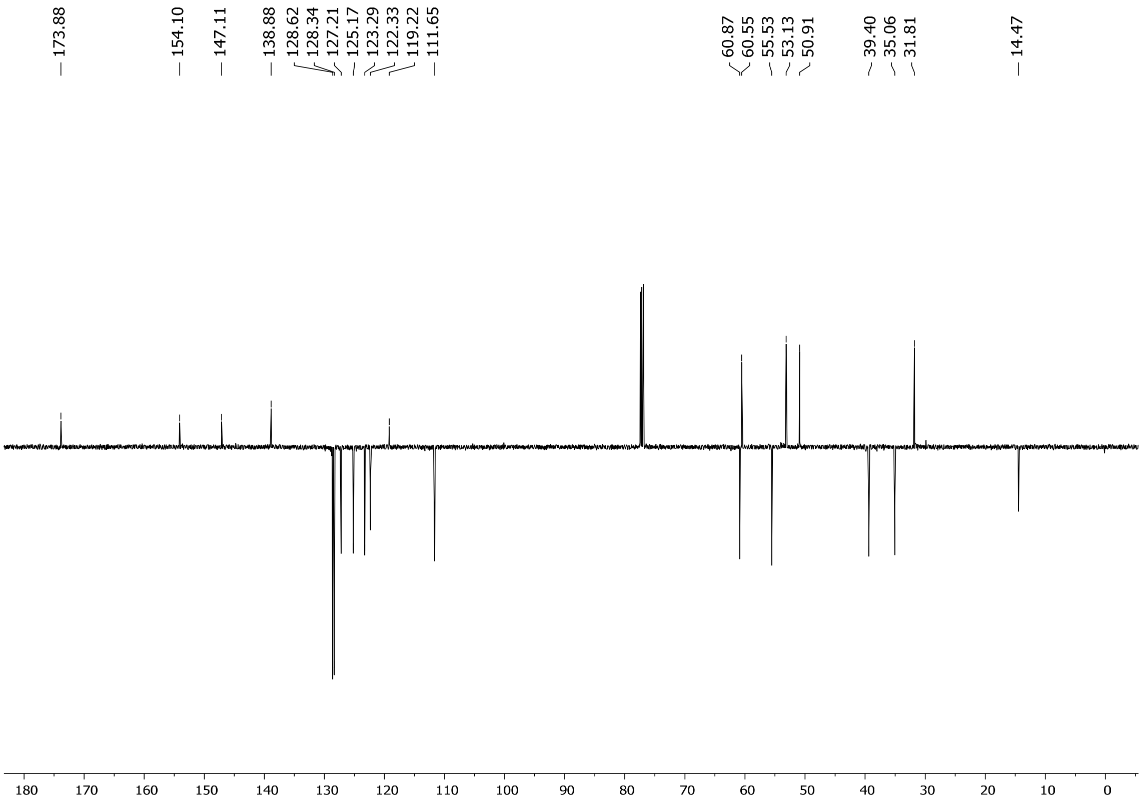
173.88
154.10
147.11
138.88
128.62
128.34
127.21
125.17
123.29
122.33
119.22
111.65
60.87
60.55
55.53
53.13
50.91
39.40
35.06
31.81
14.47
180
170
160
150
140
130
120
110
100
90
80
70
60
50
40
30
20
10
0
